# Impairment indicators for predicting delayed mortality in black sea bass (*Centropristis striata*) discards within the commercial trap fishery

**DOI:** 10.1093/conphys/coaa068

**Published:** 2020-09-08

**Authors:** Cara C Schweitzer, Andrij Z Horodysky, André L Price, Bradley G Stevens

**Affiliations:** 1 Department of Natural Sciences, University of Maryland Eastern Shore, 1 College Backbone Rd, Princess Anne, MD, 21853, USA; 2 Department of Marine and Environmental Science, Hampton University, 3 Shore Rd, Hampton, VA 23668, USA

**Keywords:** Black sea bass, discard mortality, fishing discards, RAMP, reflex impairment

## Abstract

Harvest restrictions (e.g. size, sex or species limitations) that are implemented to maintain sustainable fisheries often result in by-catch, e.g. unwanted non-target catch. By-catch is frequently discarded back into the ocean and assumed to survive. However, discarded fishes can succumb to delayed mortality resulting from accumulated stress from fishing activity, and such mortality can impede sustainability efforts. Quantifying reflex and behavioural impairments is a quick and cost-effective method to predict discard-related mortality in some species. We developed and evaluated the effectiveness of a release condition index, based on a reflex-action mortality prediction (RAMP) model, for predicting delayed mortality of black sea bass (*Centropristis striata*) caught and discarded by the commercial trap fishery in the Mid-Atlantic Bight. Accumulation of impairments, and therefore release condition index, was strongly correlated with delayed mortality of black sea bass discarded and held in sea cages. This is the first release condition index validation study to predict mortality in black sea bass and could be a useful approach for predicting delayed mortality in the commercial fishery.

## Teaser

We investigated the use of a release condition index to predict delayed mortality in black sea bass within the mid-Atlantic. Commercial trap fishing was used at the model fishery. Our results showed that a release condition index may be a good indicator of mortality.

## Introduction

### Discard mortality

A goal of sustainable management is to determine the amount of biomass that can be harvested currently in a fishery without compromising the ability of the population to regenerate. To help prevent overfishing, fisheries management imposes harvest quotas, size limitations and gear restrictions (Grafton *et al.*, 2006). Such restrictions often result in by-catch, i.e. the capturing of non-targeted species or the wrong size or sex groups of the target species. Usually, such fishes are released back into the ocean, regardless of their condition. However, discards can succumb to either immediate or delayed mortality, which can occur minutes to weeks post-release (Sainsbury *et al.*, 1993; Davis, 2002; Davis and Parker, 2004; Diamond and Campbell, 2009; Benoît *et al.*, 2013). The magnitude of discard mortality is poorly known for most species, and its underestimation can create uncertainty in stock assessments that can lead to poor management resulting in overfishing (Alverson *et al.*, 1994; Davis, 2002; Harrington *et al.*, 2005).

The US National Bycatch Report (NMFS, 2016) provides information on commercial fisheries by-catch via observer data. Unobserved and delayed mortality of discards, however, is not included in the report due to a paucity of data. Improved estimates of discard mortality are valuable for sustaining fish stocks. Fishing gear, fish handling techniques, and seasons can have profound effects on mortality rates during handling and discarding of economically valuable species (Davis *et al.*, 2001; Davis, 2002; Davis and Parker, 2004; Cook *et al.*, 2015). Traditionally, estimating delayed mortality involves tagging and recapturing large numbers of individuals, performing tag retention studies, or a combination of acoustic telemetry, captive holding studies and fishing simulation studies (Benoit *et al.*, 2012; Henderson and Fabrizio 2014; Rudershausen *et al.*, 2014; Curtis *et al.*, 2015, Lewin *et al.*, 2018). Such studies can become quite costly and time-consuming, and thus difficult to replicate. Therefore, developing and adopting an efficient and inexpensive method for effectively estimating released condition and delayed mortality can provide valuable data for stock assessment models.

Approaches to sustainability efforts for stocks are typically implemented via single species stock assessments and/or ecosystem based fisheries management, but neither takes behaviour and individual condition into account (Horodysky *et al.*, 2015, 2016; Ward *et al.*, 2016). Understanding behaviour and condition can provide insight on how environmental stimuli can influence discard mortality rates (Horodysky *et al.*, 2015), which are often species-specific.

### Black sea bass

Black sea bass (*Centropristis striata*) supports one of the most popular commercial and recreational fisheries within the Mid-Atlantic Bight (MAB), having an estimated worth of ~$6.87 million USD (NOAA 2016, 2018) for the commercial fishery alone. The commercial fishery harvests black sea bass primarily using fish traps, which account for 44% of the commercial landings for the Mid-Atlantic and Northwest Atlantic (NFSC 2017). Within the Delaware, Maryland and Virginia (Delmarva) portion of the MAB, ~5000 to 6000 active traps are fished primarily by two commercial boats.

The northern black sea bass is considered a data poor species, and there is a paucity of data regarding discard mortality. Currently, the northern black sea bass stock is no longer considered overfished and spawning stock biomass (SSB) is above biomass threshold defined by the National Oceanic and Atmospheric Administration (NOAA). Yet, despite the SSB reaching target levels in 2012, the total stock has been declining since 2013, and the SSB has been declining since 2014 (NFSC 2017). A continual decline of the black sea bass stock could be an indication that discard mortality rates are higher than what is currently estimated.

The adjusted fishing mortality (*F*) for northern and southern stocks is estimated at *F* = 0.27, which is below the target fishing mortality of *F* = 0.36 (NFSC 2017). Models evaluated in 2011 incorporated a discard mortality rate of 7% for the northern stock of black sea bass. This value was adjusted downward from a previous value of 15% (SEDAR 2011; NFSC 2017), which was based on an estimate of 13% mortality within the commercial trap fishery (Rudershausen *et al.*, 2014). However, these estimates were calculated by fishing with single traplines and soak times of 18 to 26 h, which is not common within the Delmarva region fishery, where lines of 20 traps soak for 10 to 14 days (C. Townsend, commercial trap fisherman, personal communication). Longer soaking times can result in longer spans of captivity and food depletion, which can increase stress levels, and the possibility for cage damage or injury to the fish. Furthermore, multi-trap lines can result in higher than expected mortality as a result of increased catch volume and thus increased sorting time and air exposure. It is important to have mortality data within specific regions, because fishing techniques are highly variable and region-specific. There are few published studies investigating delayed fishing mortality within the commercial black sea bass fishery, and none within the MAB.

### Impairment index: barotrauma, behaviours and reflex

The fishing process exposes fish to acute changes in temperature, pressure, and oxygen availability, as well as contact with the netting or other fish and handling that can cause severe damages or physiological stress responses, which can drastically reduce chances for survival (Davis, 2002; Cooke and Schramm 2007; Diamond and Campbell, 2009). Accumulation of physiological stress results in reflex, i.e. an immediate involuntary reaction to a stimulus, and behavioural impairments that can cause a reduction in feeding behaviour and a decreased ability to avoid predation post release (Campbell *et al.*, 2010). Reflex impairments can be quantified to estimate an individual’s condition and probable mortality through the use of reflex-action mortality predictors (RAMP; Davis, 2007). RAMP is a condition index based on scoring the presence or absence of reflex impairments and relating them to a probability of mortality. Reflex and behavioural impairments have been demonstrated as an effective way to predict discard mortality in several species including coho salmon (*Oncorhynchus kisutch*; Davis, 2007; Raby *et al.*, 2012), yellowtail founder (*Pleuronectes ferruginea*; Barkley and Cadrin, 2012; Forrestal *et al.*, 2017), red snapper (*Lutjanus campechanus*; Campbell *et al.*, 2010), bonefish (*Albula* spp.; Brownscombe *et al.*, 2015) and white sturgeon (*Acipenser transmontanus*; McLean *et al.*, 2016). However, validation experiments are required because reflex and behavioural impairments may not be effective mortality predictors in all species (Brownscombe *et al*., 2014; Gallagher *et al*., 2014; Nguyen *et al*., 2014; Struthers *et al.*, 2018).

For fish that are harvested from deeper waters, such as black sea bass, incorporating barotrauma and behavioural responses in addition to reflexes can result in more accurate predictions of probable mortality (Diamond and Campbell 2009). The effects of barotrauma, i.e. physical damage to body tissues caused by a difference in pressure, include the expansion of the swim bladder, stomach eversion, exophthalmia and intestinal protrusion. If fish are not vented or recompressed, barotrauma can increase the probability of mortality (Diamond and Campbell, 2009; Campbell *et al.*, 2010).

The adoption of RAMP scores or a release condition index (RCI) in a presence/absence manner for mortality prediction has several advantages. In addition to being quick and inexpensive, assessment protocols are minimally invasive to fish, assessments can be conducted in any environmental setting, and these methods can be taught to fishers and observers (Meeremans *et al.*, 2017). Furthermore, categorical scales to rank the robustness of behavioural impairments can result in higher rates of ambiguity for less trained individuals when compared to presence/absence analysis (Meeremans *et al.*, 2017). Such methodologies can help to rapidly obtain discard mortality rates for economically important species in both commercial and recreational fisheries, and improve overall quantity and quality of by-catch and discard data.

The purpose of this study was to (i) identify a set of testable and reliably measurable impairments for black sea bass discard, (ii) incorporate them into a release condition index, and (iii) determine the relationship between the release condition index and mortality that would allow for mortality prediction. We hypothesized that discarded black sea bass undergo delayed mortality, that such mortality increases with accumulated physiological impairments, and that mortality can be predicted using an impairment index.

## Materials and methods

### Sites and collection method

Sampling was conducted in two series at two sites that were selected based on commercial fishing activity in 2016 and 2017. Series 1 was completed in August 2017, which consisted of four fishing trips. Series 2 was completed in late October to early November 2017 consisting of two fishing trips, due to hazardous weather preventing additional outings. The sites were located ~25 km off the coasts of Delaware and Maryland in the MAB and separated by ~15 km. Depths ranged from 25 to 30 m, which are representative depth ranges for both commercial trap and recreational fishing for black sea bass. The mean air temperatures for Series 1 and Series 2 were 24.7°C ± 1.5°C SD and 17.9°C ± 3.8°C SD, respectively, during the study period. Mean bottom temperatures for Series 1 and 2 were 13.8°C ± 0.2°C SD and 18.6°C ± 2.0°C SD, respectively. The mean difference between bottom and surface temperature for Series 1 and 2 was 10.6 °C ± 0.2 °C SD and 0.7°C ± 0.1°C SD, respectively.

Fish were captured via a 384-m trapline that contained 20 standard commercial traps (1.1 × 0.53 × 0.3 m) for black sea bass (see Schweitzer *et al.*, 2018, for detailed description of trapline). Standard commercial traps are built with 3.8-cm wire square mesh and 6.7-cm diameter double circular escape vents to reduce the capture of undersized black sea bass. One trapline was deployed at each of the study sites. Traps were hauled, and fish were sorted by commercial fishers using standard fishing and sorting practices: trap buoy lines were retrieved and led over a hydraulic pinch block pulling the trap line, after which traps were emptied into a sorting bin. As the captain hauled and emptied the traps, the second fisher prepared the newly empty trap for redeployment on the stern. If the fisher was unable to completely sort through the catch and discards between traps, the next trap’s contents were emptied on top of the previous. It was commonly observed that fish of larger lengths that were more obviously legal size to fishers and fish much smaller than legal limits were sorted and discarded first, leaving fish closer to the legal-size limit to be measured and sorted or discarded last. It was atypical for a fisher to complete the sorting and discarding of a single trap’s contents before the next trap was emptied into the sorting bin.

Observations made during five additional commercial fishing trips (non-experimental) showed that ~388 s (6.5 min) were required to retrieve a complete trapline (i.e. 20 traps), for an average 19 s between each trap, and ~632 s (10.5 min) were required for sorting and discarding to be completed. Fish that fell through the sorting bin onto the deck were typically the last to be discarded. After all fish were sorted, and by-catch discarded, traps were redeployed. Standard fishing practice is to let traplines soak for 10 to 14 days after deployment. However, due to scheduling constraints, experimental traplines were left to soak for 5 days during Series 1 and 10 days during Series 2.

### Handling and assessment of discards

As part of the experimental design, undersized fish were discarded directly into a 190 L holding container filled with ~150 L of seawater. The mean water temperature within the holding container and vat was comparable to sea surface temperatures and was 24.4°C ± 0.5°C (mean ± SD) for Series 1, and 19.3°C ± 1.6°C for Series 2. Assessment of impairment began immediately on all live discards. Dead fish were not assessed. Fish were recorded as either floating or not, then were removed from the holding bin and placed on a measuring board, and total length was measured to the nearest 1 cm. Fish were then placed on a wet towel, and the fish’s head was covered for tagging. Fish were tagged with anchor T-bar Floy® tags (FD-68B; Floy Tag Company, Seattle, Washington) in the epaxial muscle beneath the dorsal fin. Impairments were selected based on the applicability to black sea bass and were assessed in a presence/absence manner adapted from Davis (2007) and Diamond and Campbell (2009), resulting in an RCI of six categorical impairments (Table 1). The processing time (i.e. length measurement, tagging and RCI assessment) ranged from ~30 to 50 s. Occasionally, fish would exhibit a catatonic state (i.e. epaxial and hypaxial muscles in a contracted state), which was documented as a loss of all reflexes and behaviours. After assessment, fish were placed in a covered 950 L insulated holding vat, 2/3 full of seawater, until processing for all fish caught at that site was complete, which ranged from ~10 to 40 min depending on the volume of catch. Fresh seawater was added to holding containers sporadically to help maintain levels of dissolved oxygen. Dissolved oxygen was monitored with a YSI PRODSS conductivity/temperature/oxygen meter and maintained at 99%.

**Table 1:** Summary of barotrauma and reflex impairments and their definitions; percent impaired is the percent of fish that exhibited each barotrauma, behaviour or reflex impairment

Impairments	Description	Classification	% Impaired *n = 625*
Floating	Fish floating ventral side up	Barotrauma	79.8
Stomach eversion	Stomach protruding out of the mouth	Barotrauma	26.9
Operculum	Inability to move the operculum	Reflex	40.5
Gag	Absence of muscle contraction when throat is stimulated by probe	Reflex	61.9
Mouth	Jaw unresponsive to manual manipulation with no muscle contraction	Behaviour	37.3
Dorsal fin	Fin does not move dorsally or caudally post-manual manipulation	Behaviour	12.8

To estimate delayed mortality rate, fish that had been scored were held in sea cages (1.2 m × 1.2 m × 0.6 m with 3.8-cm wire square mesh), which were constructed to be roughly quadruple the volume of commercial traps, and a single sea cage was attached to a single buoy line. Sea cages were placed ~460 m from each of the fishing sites. Fish were housed in sea cages for 4 days during Series 1, but 10 days during Series 2 due to hazardous weather conditions that prevented earlier retrievals. Density of fish in the cages ranged from 6 to 27 fish per cage. Sea cages were slowly retrieved by feeding the buoy line through the hydraulic pinch block. Tag numbers of fish that remained in the cage were recorded, and surviving fish were released into the ocean. The 3.8-cm wire mesh prevented fish larger than 17 cm from entering or escaping the sea cage; therefore, fish not present were presumed dead. Fish that died before RCI assessment were recorded as dead and incorporated into total discard mortality rates. No new, i.e. untagged, fish entered sea cages during the deployment period.

### Preliminary methods

A preliminary experiment was completed in May 2016, to determine the most effective impairments (Table 1) for quantifying mortality in black sea bass because indicators are species-dependent. The mean air temperature during the preliminary study was 19.6°C ± 6.4°C SD, and the mean bottom temperature was 11.2°C ± 0.4°C SD. Collection methods were identical as previously described. Handling and assessment were also conducted as previously described, except the tagging procedure, which followed Rudershausen *et al.* (2014). We used internal anchor tags (3/16″ × 9/16″ oval; Floy® tags, Seattle, Washington) that required a small incision behind the pectoral fin. Frequently, gas within the body cavity was released (i.e. venting) during the incision process, which inadvertently vented fish that exhibited barotrauma. Although venting fish improves their survival, it is not commonly done by commercial fishers; venting could artificially inflate survival estimates of fish implanted with internal anchor tags. Therefore, we used only T-bar tags for the research study in 2017 to avoid inadvertent, but inescapable venting during internal tag implantation. Since tagging procedures in 2016 differed from the 2017 sampling Series, the 2016 data were not included in the final analysis.

### Data analysis

The RCI was calculated as the sum of the number of impaired reflexes divided by 6, i.e.

$$\begin{equation*}\textrm{RCI}=(\Sigma \textrm{I})/6\end{equation*}$$

where *I* is the value (0 or 1) for any individual impairment, and the value of the RCI ranges from 0 to 1 (Table 2).

**Table 2:** Proportional mortality rates within each release condition index (RCI) bracket

RCI	*n* Impairments	*n* Fish; *n = 625*	*n* Mortality	Proportional mortality
0	0	66	5	0.08
0.17	1	106	27	0.26
0.33	2	126	43	0.34
0.50	3	137	73	0.53
0.67	4	118	66	0.55
0.83	5	66	49	0.71
1	6	6	5	0.83

Total impairment was defined as having all six impairments, resulting in an RCI of 1. Proportional mortality within each level of the RCI for fish tagged on a given date was calculated as the number of mortalities (*M_i_*) is divided by total fish (*n_i_*), where *i* indicates the number of impairments.

Statistical analyses were performed in R version 3.4.3 (R Core Team, 2016) and Prism version 7.0. A chi-square test was used to determine if mortality differed between sampling series. A Kolmogorov–Smirnov Test was used to determine if there was a difference in the frequency distribution of fish length between mortalities and survivors.

Logistic regression with a logit link was used to determine if there was a relationship between RCI and mortality for black sea bass discard and to determine if cage densities affected mortality. To determine which factors had an influence on mortality, we included barotrauma, reflexes and behaviours as separate factors within the general linear model, with fish length as a covariate. We performed a drop-in analysis of deviance test with an *F*-test link to determine the effect of each parameter. The Akaike information criterion (AIC) values from each model (*g_i_*) were used to calculate a second-order bias correction estimator (AIC*_C_*). The different models were ranked via the *Δi* values against the lowest AIC*_C_*. Model probabilities (*w_i_*) estimated the probability that a particular model was the best fitted model. Models with a *w_i_* value <0.1 were eliminated.

A conditional inference tree model was constructed using R packages ‘party’ and ‘partykit’ with mortality as the response variable and the impairments as explanatory variables (Strobl *et al.* 2009; Hothorn and Zeileis 2015). The ‘party’ package uses an adaptive learning algorithm and a statistically determined stopping criterion (i.e. a priori *P* value) to determine when branching is no longer valid. Branching criteria were determined by the deviation of the null model and set with the minimum criteria of *α* = 0.05 using Monte Carlo correction. The first node was selected based on statistical significance level compared to the six explanatory variables.

## Results

### Black sea bass discard mortality

This study used sea cages to hold RCI scored fish in order to determine the mortality rates that resulted from fishing practices. We did not observe any cage effects, such as wounds or cloudy eyes, amongst fish released after cage-holding.

**Figure 1:**
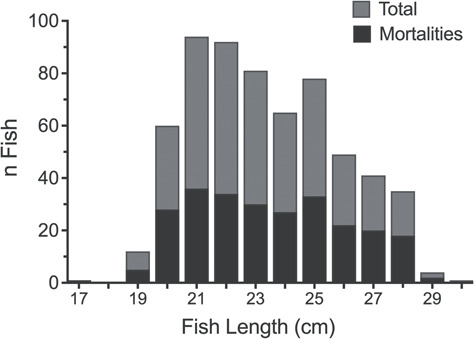
Length distribution of total catch (grey bars) and mortalities (black bars) of black sea bass discard captures within commercial traps and two research sites (*n* = 675)

The mesh size of the commercial traps and sea cages allowed black sea bass ≤ 17 cm to swim freely in and out of the trap resulting in a predominant by-catch size range of 19 to 28 cm (Fig. 1). Only a single fish was captured that was <19 cm. The minimum legal-size limit for black sea bass for commercial fisheries in the Greater Atlantic Region is 29 cm. Despite this, fishers will at times misjudge sizes and discard fish that are of legal size; because of this, we incorporated all fish that were given to us as discards including some fish of legal size (*n* = 4). There was no difference in fish length distribution between black sea bass discard mortalities and survivors (K–S test, *P* = 0.63). Discard mortality was separated into two categories: immediate and delayed. Immediate mortality was defined as discards that died before sea cage placement, which was composed of RCI assessed fish and non-assessed fish. Total discard mortality for the duration of the study was 47.1% (318/675), which included both immediate and delayed mortality. The recorded mortality rate for this study was vastly different than the mortality recorded during the preliminary study (13.7%; 20/146) where venting inadvertently occurred.

During Series 1 and 2, a total of 625 black sea bass discards were assessed for fish length, barotrauma, behavioural and reflex impairment to determine mortality. In Series 1, immediate mortality, i.e. mortality before sea cage placement, was 36.8% (160/435), and delayed mortality was 22.5% (62/273), for a total mortality of 51.0% (222/435). In Series 2, there was no immediate mortality, and delayed mortality was 38.8% (96/240). Total discard mortality was significantly higher during Series 1 than in Series 2 (*Χ*^2^*, P* = 0.007), though Series 2 had higher rates of delayed mortality (*X*^2^, *P* < 0.001). This is in contrast to the preliminary study where fish were inadvertently vented with a discard mortality of 13.7%, a similar mortality rate to the Rudershausen *et al.* (2014) study.

### RCI for mortality prediction

**Figure 2:**
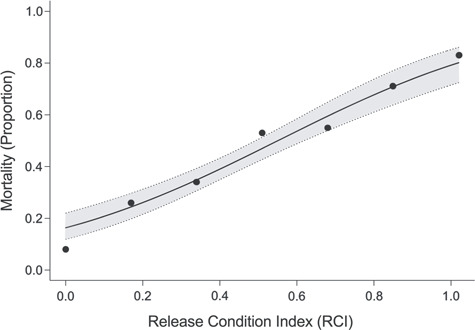
The relationship between observed release condition index (0 = no impairment; 1 = total impairment) and the proportional delayed mortality (0 = 0% mortality; 1 = 100% mortality) for black sea bass discard. Data points are plotted along a prediction function based on the g_4_ model response variable. The 95% confidence intervals are shown as the shaded area

Overall, mortality of black sea bass discard increased with the RCI (*R*^2^ = 0.97; Fig. 2). Fish exhibiting total impairment (i.e. RCI = 1.0) had a proportional mortality of 0.85, and fish with no impairment (i.e. RCI = 0.0) had a proportional mortality of 0.08 (Table 2). There was a significant difference between the mean RCI of total survivors ($\bar{x}$ = 0.35 ± 0.01 SEM) and total mortalities ($\bar{x}$ = 0.55 ± 0.02 SEM; *t* test, *P* < 0.001). The mean RCI score for discards that were placed in sea cages was significantly higher in Series 2 ($\bar{x}$ = 0.49 SEM ± 0.01) than Series 1 ($\bar{x}$ = 0.34 SEM ± 0.02; *t* test, *P* < 0.001). Furthermore, mean RCI scores were significantly higher for fish that succumbed to immediate mortality ($\bar{x}$ = 0.56 SEM ± 0.02) than for fish that were placed in sea cages ($\bar{x}$ = 0.34; t test, *P* < 0.001) during Series 1. The most frequently observed impairments were barotrauma, i.e. expansion of the swim bladder resulting in the fish floating, and the absence of the gag reflex (Table 1). Severe barotrauma, i.e. stomach eversion, was only detected in 27% of black sea bass discards.

**Table 3:** Comparison of models g_0_–g_4_ corresponding to the different variables tested for mortality prediction

Model	Variables	df	AIC*_C_*	Δ*_i_*	*w_i_*	Loglik
g_0_	M ~ 1	1	855.1	+99.9	<0.01	−426.6
g_1_	M ~ C + L	3	781.6	+26.4	<0.01	−387.8
g_2_	M ~ C	2	780.3	+25.1	<0.01	−388.1
g_3_	M ~ F + Se + D + G + Mm + O + L	8	756.8	+1.6	0.31	−370.3
**g**_**4**_	**M ~ F + Se + D + G + Mm + O**	**7**	**755.2**	**—**	**0.69**	**−370.5**

Df = degrees of freedom; AIC*_C_* = corrected AIC value; Δ*_i_* = difference between each model and the best selected model; and *w_i_* = probability that a given model provided is the best fit for the data. Loglik = log-likelihood. Variables: M = mortality; C = release condition index; L = fish length; F = fish floating; Se = stomach eversion; D = dorsal fin movement; G = gag reflex; Mm = mouth movement; O = operculum reflex. Model g_4_ was selected to be the best fit model

From the four models tested and compared against a null model, logistic regression and drop-in deviance test determined that the best fit model for probable mortality in black sea bass discard was g_4_, which included barotrauma, behaviours and reflexes, but not fish length (Table 3). Models g_1_ and g_2_ were eliminated because their probability (*w*_i_) was <0.05 and increased the AIC*_C_* by 26.4 and 25.1, respectively. Despite the AIC*_C_* increase being <2, fish length was an insignificant contributor to mortality according to the drop-in deviance test (*F* test; *P* = 0.53). Therefore, g_4_ was selected as the best fit model (Table 4). To determine if post-release cage density was a significant factor in mortality, an additional model was analyzed. Due to this model having to use a different dataset, omitting immediate mortality, the model was separated from the RCI models. Post-release cage density did not contribute to mortality (*P* = 0.83).

**Table 4:** Results from binomial (logit link) logistic modelling for the chosen model g_4_ for discarded black sea bass bycatch, including estimates, standard error (SE) and *P* values for intercept and the six impairments

RCI impairments	Estimate	SE	*P* value
Intercept	−1.93	0.27	<0.001
Floating	7.82	1.62	<0.001
Stomach eversion	4.74	1.26	<0.001
Dorsal fin	4.46	1.75	0.01
Operculum	4.05	1.11	<0.001
Gag	−3.01	1.22	0.01
Mouth	3.54	1.16	0.002

**Figure 3:**
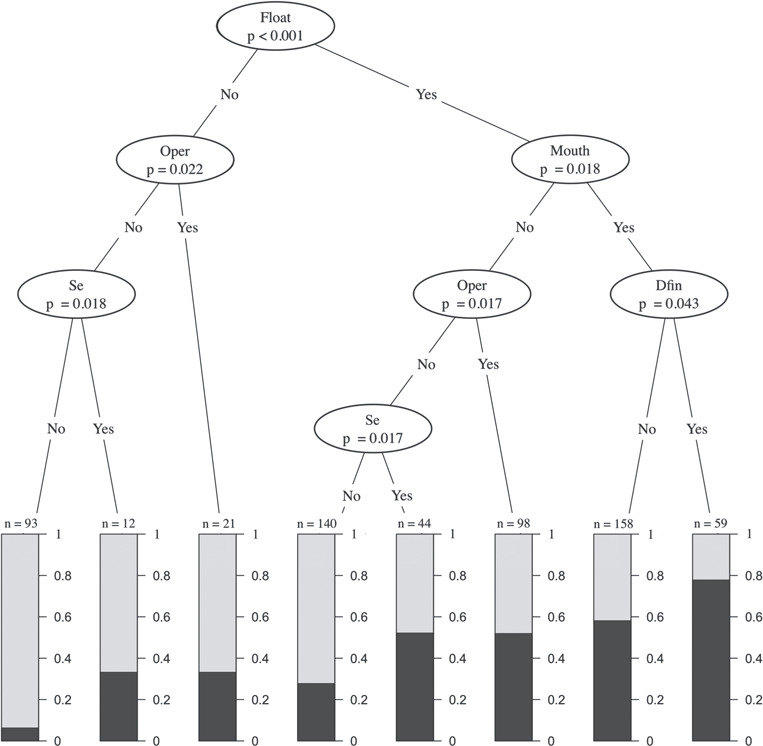
A conditional inference tree examining the impacts of the RCI score on probable mortality by using barotrauma and impairments as explanatory variables based on model g_4_. Monte Carlo corrected *P* values represent the deviation from the null hypothesis and are the base for the decision node selection. The branches are divided by ‘yes’ (impaired) or ‘no’ (unimpaired). The terminal nodes are represented by graphs where probable mortality is dark grey. Abbreviations: Float = fish floating; Se = stomach eversion; mouth = mouth movement; Oper = operculum reflex; Dfin = dorsal fish movement

To better determine how individual combinations of barotrauma, behavioural and reflex impairments affected probable mortality, a conditional inference tree was constructed from the best fit g_4_ model. Barotrauma that resulted in floating was determined to be the main splitting criteria for probable mortality. A fish just exhibiting barotrauma that results in floating has a probable mortality of ~25%. However, if a single impairment is loss of operculum movement or stomach eversion, probable mortality increases to ~35% (Fig. 3). The highest probable mortality (~80%) was estimated from two scenarios including (i) total impairment and (ii) floating and the loss of mouth and dorsal fin movement. The conditional inference tree shows up to three impairment combinations due to the set splitting criteria. This suggests that, when a fish exhibits ≥4 impairments, combinations are insignificant and the predicted model (Fig. 2) incorporating total RCI score is sufficient.

## Discussion

Our study was the first to validate an RCI for estimating delayed mortality in black sea bass, an economically valuable species along the Mid-Atlantic Bight. This study quantified barotrauma, reflexes and behavioural impairments in a presence/absence manner in order to develop a rapid and universal method for mortality prediction. An increase of impairments, and thus RCI, was strongly correlated with increases in mortality (Table 2). This research is consistent with previous reports in other species demonstrating strong correlations between accumulated impairments and mortality (Davis, 2007, 2010; Humborstad *et al.*, 2009; Campbell *et al.*, 2010; Raby *et al.*, 2012; Barkley and Cadrin, 2012; Brownscombe *et al.* 2015; Yochum *et al.*, 2015; McLean *et al.*, 2016; Forrestal *et al.*, 2017). Based on these data, we propose that the use of an RCI quantifying barotrauma, reflex loss and behavioural impairments is likely sufficient to predict delayed mortality in black sea bass discard and may be helpful as an integrated part of stock assessment (Fig. 2; Davis 2010).

A conditional inference tree may be used to increase efficiency and accuracy by distinguishing between minor and severe impairments. For instance, a fish given an RCI score of 0.17 has a predicted probable mortality of ~25% (Fig. 1); however, chance of mortality increases to ~35% if a single impairment is stomach eversion or loss of operculum movement (Fig. 3). Furthermore, a fish given an RCI score of 0.50 has a predicted probable mortality of ~45% (Fig. 2). Yet, based on the combinations of impairments, predicted mortality can increase to ~80% with floating, loss of mouth movement and loss of dorsal fin movement (Fig. 3). An RCI score of 0.33 has a predicted mortality of ~35%, yet if those two impairments are severe barotrauma (i.e. floating ventral side up coupled with stomach eversion) probable mortality increases to ~50%. Combinations of impairments appear to be an important factor for estimating mortality from lower RCI scores but are not a factor for RCI scores ≥0.67. Therefore, utilizing a conditional inference tree may improve mortality predictions for lower RCI scoring fish.

This study is not without limitations such as: (i) holding fish on deck, and (ii) holding fish within sea cages. Holding fish on deck, whilst not an uncommon practice (Campbell *et al.* 2010; Barkley and Steven 2012; Benoît *et al.* 2012; Sulikowski *et al.* 2018), may, however, create a bias by keeping some fish at surface pressure longer than would normally happen during commercial fishing practices. While forced recompression can reverse the visible signs of barotrauma (Parker *et al.* 2006), prolonged exposure to surface pressure and temperature may exacerbate the stress response in black sea bass (downward bias), extending the time for recovery; alternately, surface holding may serve as a protectant from predation (upward bias) for positively buoyant fish (Brownscombe *et al.* 2013). Within our study, fish were held in sea cages for 4 days during Series 1 and 10 days during Series 2 to determine mortality that directly resulted from the fishing process. The use of sea cages is a widely used method that permits tracking of nearly all fish assessed for the RCI with minimal adverse effects or no significant effects on mortality rates (Gitschlag and Renaud 1994, Jarvis and Lowe 2008, Hannah *et al.* 2012, Sulikowski *et al.* 2018). Trade-offs for fish retention in sea cages included (i) their provision of protection from predation (upward bias), (ii) restriction of food availability (downward bias) and (iii) possibility of a cage effect (downward bias). Elimination of predation probability resulted in increased survival by limiting access of predators to recovering black sea bass. Food restriction could become an issue if individual fish that did not survive were preyed upon by surviving fish as evident of skeletal remains found in cages. Due to scheduling limitations with fishers, commercial traps were soaked for 4 days during Series 1. A shorter soak time for fish traps (4 days) could decrease the risk for mortality compared to longer soak durations, which are commonly practiced. However, in Series 2, due to hazardous weather conditions, we were unable to retrieve fish from sea cages for 10 days. Even though a 10-day trap soak duration resembled the more standard practice, a longer sea cage holding could have contributed to mortality.

Observations of effects on mortality from hazardous sea conditions and longer sea cage duration can be valuable. Traps are typically soaked for 10 days, but during hazardous weather conditions, traps may not be retrieved for up to 20 days (C. Townsend, commercial trap fisherman, personal communication). These observations raise the questions:

1) What is the pertinent timeline for impairment-related delayed mortality? Additional experiments could increase sea cage observations in increments of 2 or 3 days extending out for 10 days to develop a mortality curve.

2) Do longer trap soak times during hazardous weather conditions result in higher delayed mortality of discards? Acquiring more data on RCI assessments during post-hazardous sea weather conditions and prolonged soak times is needed to assess weather effects, which can help further develop the conditional inference tree model.

Overall, in Series 1, we observed a higher mortality (50.8%), but a lower delayed mortality (22.9%). This could be an effect of mortality occurring at a more rapid rate during warmer months, where the survival curve could plateau at an earlier time point, whereas mortality is a more delayed occurrence during cooler months. Fish that succumbed to immediate mortality had on average significantly higher RCI score ($\overline{\underset{\_}{x}}$ = 0.58) than fish placed in sea cages ($\overline{\underset{\_}{x}}$ = 0.34) within Series 1; however, fish placed in sea cages within Series 2 had on average higher RCI scores ($\overline{\underset{\_}{x}}$ = 0.49), which also exhibited higher delayed mortality (39.7%), but overall lower total mortality. Immediate mortality was not observed in Series 2, which implies that higher temperatures during Series 1 may have contributed to an earlier onset of mortality, whereas cooler air temperatures during Series 2 may have prolonged the onset of mortality contributing to higher rate of delayed mortality. Similar observations in changes in mortality rate between seasons have been reported in other species (Muoneke 1992; Cooke and Suski, 2005; Crossin *et al.*, 2007; Crossin *et al.*, (2008); Cicia *et al.*, 2012; Curtis *et al.*, 2015), which could be a direct result of temperature shock between changes of water and air temperature. However, more research is needed to obtain seasonal differences in RCI scores and to obtain longitudinal mortality data to determine a mortality curve for black sea bass.

We observed total black sea bass discard mortality to be 47.1% under the conditions for this research study, much greater than the mortality estimates presently used by NOAA (i.e. *F* = 0.27 mortality), and Standardized Bycatch Reporting Methodology (SBRM; 15% mortality; NFSC 2017), which is based on commercial mortality rates reported by Rudershausen *et al.* (2014; i.e. 14%). Differences between previous mortality reports and our findings could be due to fishing technique, time of data acquisition and differences in tagging procedure. Rudershausen *et al.* (2014) encompassed aspects of both commercial and recreational fishing, so study comparisons are limited to the commercial portion of that study. Their trap fishing methods consisted of researchers hauling traps on a single trap line and sorting fish, rather than having professional fishermen haul a complete trapline under actual fishing conditions. It is possible that researchers may handle fish more cautiously compared to fishers, which could reduce fish stress and mortality. The use of single traplines might be representative of techniques within that study region but are not representative of multi-trap gear used by commercial fishermen within the Delmarva region, due to a requirement to reduce the number of vertical buoy lines in the water. Additionally, Rudershausen *et al.* (2014) reported the observation of gas escaping during the internal tagging process. We observed similar occurrences during our preliminary experiment when accidentally puncturing the body cavity whilst using internal anchor tags; consequently, we observed a similar mortality rate (13.7%). When correcting for inadvertent venting, the authors concluded that their observed mortality rate did not differ when compared to the mortality rate of non-tagged fish. We, however, observed a substantial increase in mortality when venting was eliminated due to the use of T-bar tags. Venting may have more of an effect when fish are also exposed to longer emersion periods or air exposure (e.g. few seconds to >10.5 min) and possibly more aggressive handling techniques. Emersion times are dependent on the fisher’s sorting experience and catch volume, which can drastically affect emersion periods and therefore mortality rates. Therefore, it is crucial to obtain mortality data from several different regions and under typical fishing practices since our data and that of Rudershausen *et al.* (2014) suggest that mortality rates can fluctuate substantially depending on fishing technique.

We found that 82.7% of fish exhibit some variant of barotrauma (floating and/or stomach eversion; Table 1), although floating due to barotrauma is not always immediate. When fish are discarded immediately back into the ocean, some discards have the ability to descend resulting in self-recompression. Similar results were reported by Rudershausen *et al.* (2014). Since sorting times ranged from a few seconds to 10.5 min, a subset of discarded fish would have possessed the ability to swim towards the ocean bottom and self-compress before succumbing to buoyancy issues and floating ventral side up took effect. However, no fish discarded towards that latter part of sorting had the ability to self-recompress. Since fish were held in an insulated vat until sea cage placement, fish that may have had the ability to recompress themselves succumbed to floating ventral side up before sea cage placement. To partially account for this, fish underwent forced recompressed in sea cages. We hypothesized that this would compensate for individuals discarded early and therefore possessing the ability to re-recompress themselves during standard fishing practices. We were unable to quantify the variability in which black sea bass lose the ability to self-recompress, or the duration of its effect without altering the fishing process or RCI assessment. Future experiments could focus on: (i) using the RCI in tag and release studies to determine if the time of self-recompression reduces mortality, and (ii) obtaining data focusing on optimal emersion periods for survival and to prevent floating from barotrauma, which could be important for making recommendations to adjust fishing technique in order to help reduce delayed mortality.

It is important to update estimates of discard mortality, as changes in stock parameters and biological reference points, weather, ocean conditions and even changes in personnel and experience can profoundly affect discard mortality rates. It is not uncommon for deckhands to change several times within a season (C. Townsend, commercial fisher, personal communication), which can increase fish sorting and handling times, and therefore air exposure. A better understanding of the variability of mortality rates could help in creating more accurate stock prediction models. A systematic and cost-effective approach for estimating discard-related mortality, such as utilizing an RCI, could allow for a more rapid and universal method for estimating delayed mortality within the black sea bass fishery.

To better understand and ultimately estimate delayed mortality of discards, data need to be collected under a variety of conditions (e.g. seasons, emersion time, fishing gear, depth) that are representative of the fishing season and fishing procedures for the species of interest and region. Traditional methods for estimating delayed mortality can be costly and time-consuming, restricting the data collection to funded institutions and scientifically trained individuals. By contrast, using an RCI can considerably reduce cost and time for data collection that is adaptable to commercial and recreational fisheries and is easily conducted by researchers, fishery observers and citizens with minimal training. Future researchers can apply the RCI to estimate delayed mortality in black sea bass within both commercial and recreational fisheries, and under variations in environmental conditions and handling procedures to better understand mortality rates. This information can be used by stakeholders to develop more efficient fishing and handling practices that result in the lowest probable mortality of discarded by-catch whilst not substantially altering fishers catch per unit effort (CPUE). Our data can also be used to improve management models, and make recommendations to policy in order to maintain sustainability of the black sea bass fishery.

## Funding

This project was supported by the National Oceanic and Atmospheric Administration (NOAA) Living Marine Resources Cooperative Science Center [NA16SEC4810007]. AZH also received support from NSF #1911928.
